# Hepatocellular carcinoma with characteristic mucin production: a case report

**DOI:** 10.4076/1757-1626-2-6789

**Published:** 2009-08-25

**Authors:** Kyung Hwa Lee, Young Bog Kim, Sung Bum Cho, Min Cheol Lee, Chang Soo Park, Jae Hyuk Lee

**Affiliations:** 1 Department of Pathology, Seonam University, College of Medicine#720, Gwangchi-dong, Namwon, Jeollanamdo, 590-711Republic of Korea; 2 Department of Surgery, Seonam University, College of Medicine#720, Gwangchi-dong, Namwon, Jeollanamdo, 590-711Republic of Korea; 3 Department of Internal Medicine, Chonnam National University Medical School#5, Hak-dong, Dong-gu, Gwangju, 501-749Republic of Korea; 4 Department of Pathology, Chonnam National University Medical School#5, Hak-dong, Dong-gu, Gwangju, 501-749Republic of Korea

## Abstract

We present a unique case of hepatocellular carcinoma with mucin-producing gland formation. A 53-year-old man with hepatitis B infection presented with weight loss for the past month. Computed tomography demonstrated a 10 × 9.8 cm mass in the right hepatic lobe accompanied by cirrhotic changes in the hepatic parenchyma. Right hepatectomy was performed, and the tumor cut surface showed a poorly-circumscribed, white to pink tumor with numerous nodules and extensive necrosis. Microscopically, the tumor was composed of thick trabeculae and large, irregularly-shaped islands, both of which were filled with pleomorphic eosinophilic hepatoid cells or gland-forming columnar cells with mucin production. Those cells were immunoreactive for cytokeratin 19 in both the trabeculae and the glands. In some tumor cells, limited immunoreactivity for cytokeratin 7, epithelial membrane antigen and carcinoembryonic antigen was noted. The cells forming thick trabeculae were focally positive for hepatocyte paraffin 1 and alpha-fetoprotein. We suggest that this tumor shows bidirectional differentiation into hepatocytes and cholangiocytes, supporting the concepts that human hepatocarcinogenesis can be based on transformation of progenitor cells which can imply divergent differentiation.

## Introduction

The majority of the primary liver cancers can be classified histologically as either hepatocellular carcinoma (HCC) or cholangiocarcinoma (CC). Combined hepatocellular and cholangiocarcinomas (CHCs) account for only a small portion, with varying incidence up to 6.3% [1,2]. CHC is defined as a tumor containing unequivocal elements of both hepatocellular and cholangiocarcinoma [3]. The diagnostic criteria of CHCs remain still unclear, reflecting the heterogeneity of the histologic features of CHCs. Their existence has provided controversies on the hepatocarcinogenesis. We present a unique case of primary liver carcinoma with dual phenotype of hepatocytes and cholangiocytes.

## Case presentation

**Figure 1.**
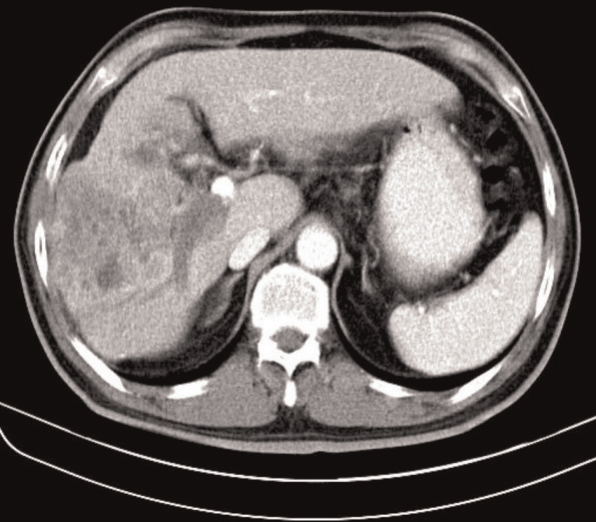
Computed tomography scan of the abdomen shows a large liver mass with heterogeneous enhancement in the right hepatic lobe.

A 53-year-old Korean male presented with a liver tumor detected at another hospital during check-up for weight loss for the past month. His past medical history revealed that he had been a hepatitis B (HB) virus carrier. His family history was noncontributory. Serologic examination on admission was positive for HB surface antigen, and negative for HB surface antibody, hepatitis C virus, hepatitis A virus, and human immunodeficiency virus. The indocyanine green retention rate at 15 min (ICGR_15_) was 26.5%. Other laboratory tests revealed elevated levels of serum alkaline phosphatase (137 IU/L), carcinoembryonic antigen (CEA, 9.42 ng/ml), alpha-fetoprotein (AFP, 93.2 IU/ml), carbohydrate antigen 19-9 (CA19-9, 500.8 U/ml), and aspartate transaminase (AST, 54 IU/L). Blood analysis, serum electrolytes, and other hepatic function tests were within the normal ranges. His blood glucose level on admission was as high as 652 mg/dl, but had been well controlled by oral hypoglycemic agents during his hospital stay. Computed tomography (CT) scan showed a 10 × 9.8 cm heterogeneously enhancing mass in the right hepatic lobe (Figure 1). The lesion was accompanied by cirrhotic changes in the hepatic parenchyma and tumoral thrombosis in the right portal vein. Right hepatectomy with cholecystectomy was performed. The tumor was described by the surgeon to involve the whole right hepatic lobe. Two weeks postoperatively, CT scan displayed substantial regrowth of the residual masses (3.2 cm and 1 cm, respectively). The patient underwent transcatheter arterial chemoembolization. However, he developed uncontrolled gastric ulcer bleeding and hepatic encephalopathy. He died three months after the surgery.

**Figure 2.**
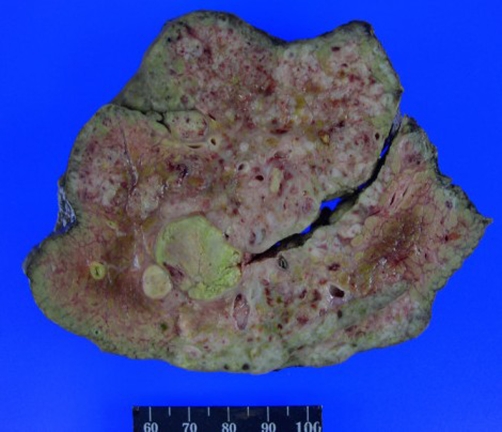
The tumor cut surface displays a poorly circumscribed, white to pink mass with extensive necrosis.

**Pathological findings** After right hepatectomy, a 16 × 11 × 11 cm liver specimen weighing 875 g was submitted. The tumor cut surface showed a poorly-circumscribed, firm, white to pink tumor with numerous nodules (Figure 2).

**Figure 3.**
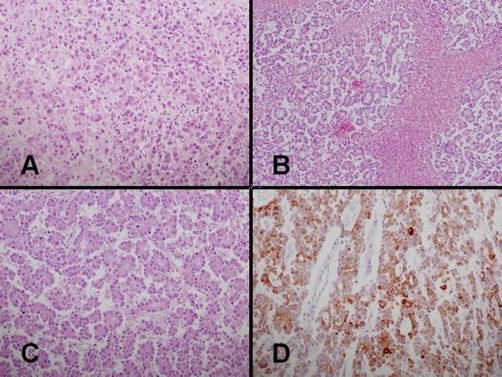
**(A)** Hepatoid tumor cells showing eosinophilic cytoplasm and moderate nuclear atypism (Original magnification × 200, H&E stain). **(B)** Loosely arranged glandular structures intermingled with geographic necrosis (Original magnification × 100, H&E stain). **(C)** Columnar cells forming regular glands without desmoplasia (Original magnification × 200, H&E stain). **(D)** Immunoreactivity of both the trabecular portion and the glandular portion for CK19 (Original magnification × 200, avidin-biotin-peroxidase stain).

Microscopic examination revealed a predominantly HCC with a widespread permeation to the surrounding cirrhotic parenchyma. Typical HCC lesions showed both solid and trabecular patterns with extensive geographical necrosis. Some of the tumor cells revealed hepatoid features, i.e. abundant eosinophilic cytoplasm and moderate nuclear atypism (Figure 3A). In a larger portion, tumor cells showed a unique growth pattern in which thick trabeculae or irregularly shaped tumor islands were filled with compact or loose mucin-producing glands (Figure 3B). Glandular portions consisted of relatively regular columnar cells without intervening desmoplastic stroma. The columnar cells had hyperchromatic nuclei in the abluminal side and moderate amount of eosinophilic cytoplasm with the formation of apical microvilli (Figure 3C). Some of those cells contained abundant intracytoplasmic mucin, creating signet-ring cell features. The gland-filled areas were occupying more than 70% of tumor components, which was confirmed with periodic acid-Schiff staining after diastase treatment. Bile production was not seen. In the borders of cirrhotic nodules small uniform cells were organized in clusters, sometimes forming flattened tubular structures. Proliferative biliary epithelia with tubule-formation or mucin-production were easily found between cirrhotic nodules near larger bile ducts.

For immunohistochemical studies, paraffin-embedded tissue was stained using the avidin-biotin peroxidase complex method. Tissue sections were incubated with primary antibodies against cytokeratin (CK)7, CK19, CK20, AE1, AE3, CEA, epithelial membrane antigen (EMA), AFP, hepatocyte paraffin 1 (HepPar1), c-kit, CD34, synaprophysin. The antibodies used are summarized in the table (Table 1). Tumor cell cytoplasm in both the trabecular and the glandular areas showed diffuse strong immunoreactivity for CK19 (Figure 3D). In some glandular portions, limited positivity for CK7, EMA and CEA was noted. Small tubule-forming cells at the margins of cirrhotic nodules were highlighted by CK7 and CK19 staining but only a few cells were positive for HepPar1. The cells forming thick trabeculae were focally positive for HepPar1 and AFP. Those antibodies known as hepatic progenitor markers, i.e., c-kit, CD34, chromogranin-A could not detect positively stained cells. These immunohistologic features confirmed the dual phenotypic differentiation of the present tumor into both hepatocytes and biliary epithelium.

**Table 1.** Antibodies used and immunohistochemical results

Specificity	Clones	Dilution	Pretreatment	Manufacturer	Results			
					Tumor		Cirrhotic nodule	
					Trabeculae	Glands	Tubule	Hepatic cord
CK7	OV-TL 12/30	1:200	pepsin	Dako, Glostrup, Denmark	+*	+	++	+
CK19	RCK108	1:200	pepsin	Dako	++	++	+	-
CK20	Ks20.8	1:200	pepsin	Dako	++	++	-	-
AE1	AE1	2× (predilute)	pepsin	Zymed, San-Francisco, CA	++	++	+	-
AE3	AE3	1:200	pepsin	Zymed	-	-	-	-
CEA	polyclonal	1:200	pepsin	Zymed	+	+	-	-
EMA	E29	1:400	pepsin	Dako	+/-	+	+	-
AFP	ZSA06	1:200	pepsin	Zymed	+	-	-	-
HepPar1	OCH1E5	1:150	microwave	Dako	+	-	+/-	-
c-kit		1:150	microwave	Dako	-	-	-	-
CD34	QBEnd 10	1:400	pepsin	Dako	-	-	-	-
Chromogranin-A	DAK-A3	1:200	microwave	Dako	-	-	-	-

* Staining intensity: ++, strong; +, weak; -, absence.

## Conclusions

CHC is a rare primary liver carcinoma containing both elements of hepatocellular and cholangiocarcinoma [3]. Clinically, CHC has overlapping features with HCC; hepatic cirrhosis and common viral markers are often positive, and the AFP level is frequently elevated. Taguchi et al. described clinical features of CHCs different from those of ordinary HCC [1]. Serum levels of AFP were relatively low in most cases. The positive rate and serum level of CEA was also low. Although CHC is more closely related to HCC than to CC, it follows a more aggressive clinical course than that of ordinary HCC. Each researcher, however, reported various incidences of hepatitis and cirrhosis due to differing etiology depending on reporting areas [1,2].

Despite several trials on the classification of CHCs, its diagnostic features still remain controversial. Of those classifications, a widely accepted classification by Taguchi et al. is as follows: 1) type I, in which each area consisting of HCC or CC is clearly separable; 2) type II, in which the HCC and CC areas are contiguous with an intervening area of transition; and 3) type III, in which the tumor was not readily classifiable as HCC and CC, and composed of tumor cells showing morphological features intermediate between HCC and CC [1]. The above definition of CHC by World Health Organization (WHO) apparently excludes those tumors in which either forms of growth are insufficiently differentiated for positive identification [3]. Many liver tumors with intermediate features, anyhow, comprise significant portion of CHCs, and their diagnosis remains challenging. Several cases equivalent to the type III by Taguchi et al. have been reported and described as a tumor comprised of small cells with a phenotype intermediate between hepatocytes and cholangiocytes [4,5]. Robrechts et al. described “primary liver progenitor cell tumor” as a monomorphic population of small cells with scant cytoplasm and a trabecular architecture, with an immunophenotype intermediate between that of hepatocytes and that of biliary epithelium [4], while Theise et al. reported “stem cell tumors” as a mixed population of oval cells dedifferentiating into both hepatocytic and biliary type epithelia [5].

The present case seemed to be only composed of HCC at first glance. But the majority of the tumor was thought to be the biphenotypic components with tumor growth pattern of HCC and mucin-containing columnar cells forming true glands. Glandular portions were compatible with CC, for the tumor cells were positive for biliary epithelial makers and producing mucin. The mucin-producing glands in the thick trabeculae and irregularly shaped islands had unique features, which have been hardly reported as a part of features in HCC. Their morphological phenotype was different from that of typical CC, in that these glands were located in the seemingly HCC-like trabeculae and clusters without desmoplastic changes. And the present case is definitely different from the pseudoglandular or pseudoacinar variant of HCCs, for the latter shows complete negative reaction in mucin staining [3]. The present case is also different from the previously reported ‘primary liver tumor of intermediated phenotype’, in that this tumor was not composed of the small uniform tumor cells. We suggest that the present case is a primary liver carcinoma with dual phenotypes of HCC and CC, supporting the concepts that human hepatocarcinogenesis may be based on transformation of progenitor cells which can imply divergent differentiation.

Three explanations of the histogenesis of CHC were suggested parallel to the classification: as a collision or intermingling of two independent primary tumors; as the transformation of a primary HCC or CC into the other; and as differentiation from a bipotential hepatic progenitor cell into morphologically different malignant components [5]. Recent studies confirm the existence of a hepatic progenitor cell population in humans within the liver, though their role in human hepatocarcinogenesis has not been confirmed [6].

Wu et al. have reported that approximately 30% of morphologically pure HCCs showed immunohistochemical evidence of biliary differentiation [7]. In animal models of hepatocarcinogenesis there are proliferations of small, undifferentiated, oval shaped cells which are referred to as ‘oval cells’ [6]. These cells are thought to be facultative bipotential progenitor cells (or stem cells) and to be related to the development of hepatocellular malignancies. Although more elaborate explanation of the hepatocarcinogenesis is needed, data accumulated so far support the hypothesis of carcinogenesis from hepatic progenitor cells with a dual potential. The small tubule-forming cells around dysplastic nodules in the present case were pretty similar to the ‘oval-like’ cells, as were the ‘transitional’ cells described by Theise *et al* [5]. As described in the cases of Theise *et al*. [5], the undifferentiated cells merge with hepatocytes in the cirrhotic nodules, and are contiguous with proliferative biliary epithelium in the surrounding fibrous connective tissue, suggesting both the hepatocytic and biliary components are arising from the undifferentiated population.

There are no markers that are absolutely indicative of stem cells in the liver. While c-kit has been proved to be suggestive in some work, chromogranin-A and CD34 have been reported to be expressed by human hepatic stem cells [8]. Previous work has demonstrated that hepatoblasts in human livers co-express CK19 and the antigen recognized by monoclonal antibody HepPar1 [9]. In the present case, the small oval-shaped cells were positive for CK19, but showed only limited immunoreactivity for HepPar1. Even if the oval-shaped cells with monotonous features in this case were not the exactly same hepatic progenitor cells, they can be considered to display in-between stages from undifferentiated progenitor cells to differentiated hepatocytes.

Immunohistological stains for CK have been considered to be useful to distinguish HCC and CC, for CK expression is believed to be preserved during the neoplastic transformation. Specifically, HCC was positive for CK8, CK18, and CC was positive for CK7 and CK19 in addition to the former CKs [8]. Tickoo et al. suggested that CC-like areas to be more positive for CK7 with a higher tendency for immunoreactivity for CK19 than HCC-like areas [2]. Absolute dependence on these markers may at times be diagnostically misleading.

Using in situ hybridization for albumin messenger ribonucleic acid (mRNA), a sensitive and specific hepatocellular marker, Tickoo et al. confirmed biphenotypic differentiation of CHCs after demonstrating positivity for both hepatocellular (albumin mRNA) and biliary CK markers [2]. D’Errico et al. have reported focal positivity for albumin mRNA in four of six peripheral CCs, as well as in biliary cystadenocarcinomas even without the morphological evidence of hepatocellular carcinoma differentiation in these cases [10]. Even the border between pure HCCs and pure CCs could be blurred by the immunohistochemical assessment on the phenotype expression. More supportive studies will be needed in the future. These results may indicate primary liver tumor with various phenotypic expressions could originate from hepatocytic progenitor cells.

## Abbreviations

AFP: alpha-fetoprotein
AST: aspartate transaminase
CA: carbohydrate antigen
CC: cholangiocarcinoma
CD: cluster of differentiation
CEA: carcinoembryonic antigen
CK: cytokeratin
CHC: combined hepatocellular and cholangiocarcinoma
CT: computed tomography
EMA: epithelial membrane antigen
HB: hepatitis B
HCC: hepatocellular carcinoma
HepPar1: hepatocyte paraffin 1
ICGR_15_: indocyanine green retention rate at 15 min
mRNA: messenger ribonucleic acid

## References

[bib-001] Taguchi J, Nakashima O, Tanaka M, Hisaka T, Takazawa T, Kojiro M (1996). A clinicopathological study on combined hepatocellular and cholangiocarcinoma. J Gastroenterol Hepatol.

[bib-002] Tickoo SK, Zee SY, Obiekwe S, Xiao H, Koea J, Robiou C, Blumgart LH, Jarnagin W, Ladanyi M, Klimstra DS (2002). Combined hepatocellular-cholangiocarcinoma: a histopathologic, immunohistochemical, and in situ hybridization study. Am J Surg Pathol.

[bib-003] Wittekind C, Fischer HP, Ponchon T, Hamilton SR, Aaltonen LA (2000). Combined hepatocellular and cholangiocarcinoma. Pathology and Genetics of Tumours of the Digestive System.

[bib-004] Robrechts C, De Vos R, Van den Heuvel M, Van Cutsem E, Van Damme B, Desmet V, Roskams T (1998). Primary liver tumour of intermediate (hepatocyte-bile duct cell) phenotype: a progenitor cell tumour?. Liver.

[bib-005] Theise ND, Yao JL, Harada K, Hytiroglou P, Portmann B, Thung SN, Tsui W, Ohta H, Nakanuma Y (2003). Hepatic ‘stem cell’ malignancies in adults: four cases. Histopathology.

[bib-006] Sell S (1993). The role of determined stem-cells in the cellular lineage of hepatocellular carcinoma. Int J Dev Biol.

[bib-007] Wu PC, Fang JW, Lau VK, Lai CL, Lo CK, Lau JY (1996). Classification of hepatocellular carcinoma according to hepatocellular and biliary differentiation markers. Clinical and biological implications. Am J Pathol.

[bib-008] Yamamoto T, Uenishi T, Ogawa M, Ichikawa T, Hai S, Sakabe K, Tanaka S, Kato H, Mikami S, Ikebe T (2005). Immunohistologic attempt to find carcinogenesis from hepatic progenitor cell in hepatocellular carcinoma. Dig Surg.

[bib-009] Haruna Y, Saito K, Spaulding S, Nalesnik MA, Gerber MA (1996). Identification of bipotential progenitor cells in human liver development. Hepatology.

[bib-010] D'Errico A, Deleonardi G, Fiorentino M, Scoazec JY, Grigioni WF (1998). Diagnostic implications of albumin messenger RNA detection and cytokeratin pattern in benign hepatic lesions and biliary cystadenocarcinoma. Diagn Mol Pathol.

